# Author Correction: The bromodomain containing protein BRD-9 orchestrates RAD51–RAD54 complex formation and regulates homologous recombination-mediated repair

**DOI:** 10.1038/s41467-022-35058-y

**Published:** 2022-11-23

**Authors:** Qin Zhou, Jinzhou Huang, Chao Zhang, Fei Zhao, Wootae Kim, Xinyi Tu, Yong Zhang, Somaira Nowsheen, Qian Zhu, Min Deng, Yuping Chen, Bo Qin, Kuntian Luo, Baohua Liu, Zhenkun Lou, Robert W. Mutter, Jian Yuan

**Affiliations:** 1 Department of Oncology, Mayo Clinic, Rochester, MN 55905 USA; 2 Department of Radiation Oncology, Hubei Cancer Hospital, Tongji Medical College, Huazhong University of Science and Technology, Wuhan, 430022 China; 3 Mayo Medical Scientist Training Program, Mayo Clinic School of Medicine and Mayo Clinic Graduate School of Biomedical Sciences, Mayo Clinic, Rochester, MN 55905 USA; 4 Department of Molecular Pharmacology and Experimental Therapeutics, Mayo Clinic, Rochester, MN 55905 USA; 5 Research Center for Translational Medicine, East Hospital, Tongji University School of medicine, Shanghai, 200120 China; 6 Key Laboratory of Arrhythmias of the Ministry of Education of China, East Hospital, Tongji University School of Medicine, Shanghai, 200120 China; 7 Guangdong Key Laboratory of Genome Stability and Human Disease Prevention, Department of Biochemistry & Molecular Biology, School of Basic Medical Sciences, Shenzhen University Health Science Center, Shenzhen, 518055 China; 8 Department of Radiation Oncology, Mayo Clinic, Rochester, MN 55905 USA

**Keywords:** Biochemistry, Cell biology, Molecular biology

Correction to: *Nature Communications* 10.1038/s41467-020-16443-x, published online 26 May 2020

The original version of this Article contained an error in Fig. 2a, in which the RAD51, RAD54 and MERGE panels displaying representative images for BRD9 shRNA1 were from an incorrect sample. This has been corrected in both the PDF and HTML versions of the Article. The Source Data file has also been replaced to include the correct uncropped images for Fig. 2a.

