# Supplementary material for: The bromodomain containing protein BRD-9 orchestrates RAD51–RAD54 complex formation and regulates homologous recombination-mediated repair
Source: Nat Commun. 2020 May 26;11:2639. doi: 10.1038/s41467-020-16443-x (PMC7251110; doi:10.1038/s41467-020-16443-x)
Supplement: Supplementary file 1 — Supplementary Information [file 41467_2020_16443_MOESM1_ESM.pdf]

The Bromodomain Containing Protein BRD-9 Orchestrates RAD51-RAD54 Complex formation and Regulates Homologous Recombination-mediated repair

Supplementary Information (Supplementary Figure1-10)

Zhou et al

Supplementary Figure 1

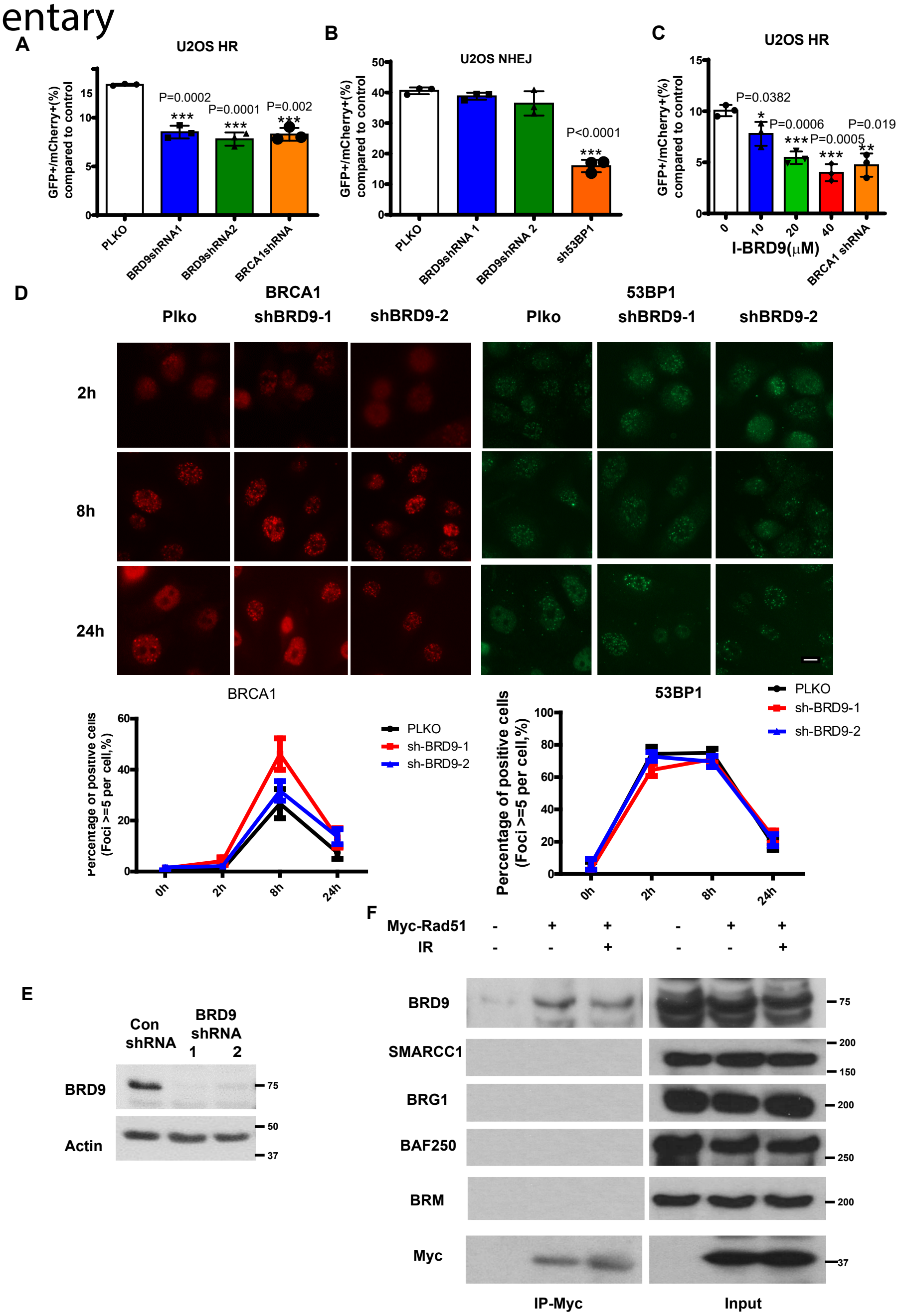

Supplementary Figure1. BRD9 is Required for HR but not NHEJ activity.

(A-B) U2OS cells were infected with the indicated shRNAs and reporter constructs. HR (A) and NHEJ (B) assay was performed Shown are the representative data (mean ± SEM) from n=3 biologically independent samples. ***p<0.001 by 2-sides unpaired t-test.

(C) OVCAR8 cells expressing the HR reporter and indicated shRNAs were exposed to the indicated doses of BRD9 inhibitor. 24hrs later, HR capacity was assessed by flow cytometry. Shown are the representative data (mean ± SEM) from n=3 biologically independent samples. *p<0.05, **p<0.01, ***p<0.001 by two-sided unpaired t-test.

(D) OVCAR8 cells were infected with lentivirus expressing control (Ctrl) or BRD9 shRNA. Cells were exposed to 2Gy irradiation and fixed at the indicated time points. Cells were stained for the indicated foci. Shown are the representative images of BRCA1 and 53BP1 foci after the indicated treatments and indicated time following 2Gy irradiation. BRCA1 and 53BP1 foci in OVCAR8 cells after the indicated treatment and time following 2 Gy irradiation were quantified. Shown are the representative data (mean ± SEM) from three independent experiments. Scale bar, 10 µm.

(E) OVCAR8 cells were infected with lentivirus expressing control (Ctrl) or BRD9 shRNA. Lysates were collected for Western blot. Blots were probed with the indicated antibodies.

(F) 293T cells were transfected with Myc-tagged RAD51. 48h after transfection, cells were exposed to 10Gy irradiation. Lysates were collected after 8 hours. Immunoprecipitation with anti-Myc beads was performed. Blots were probed with the indicated antibodies..

Supplementary Figure 2

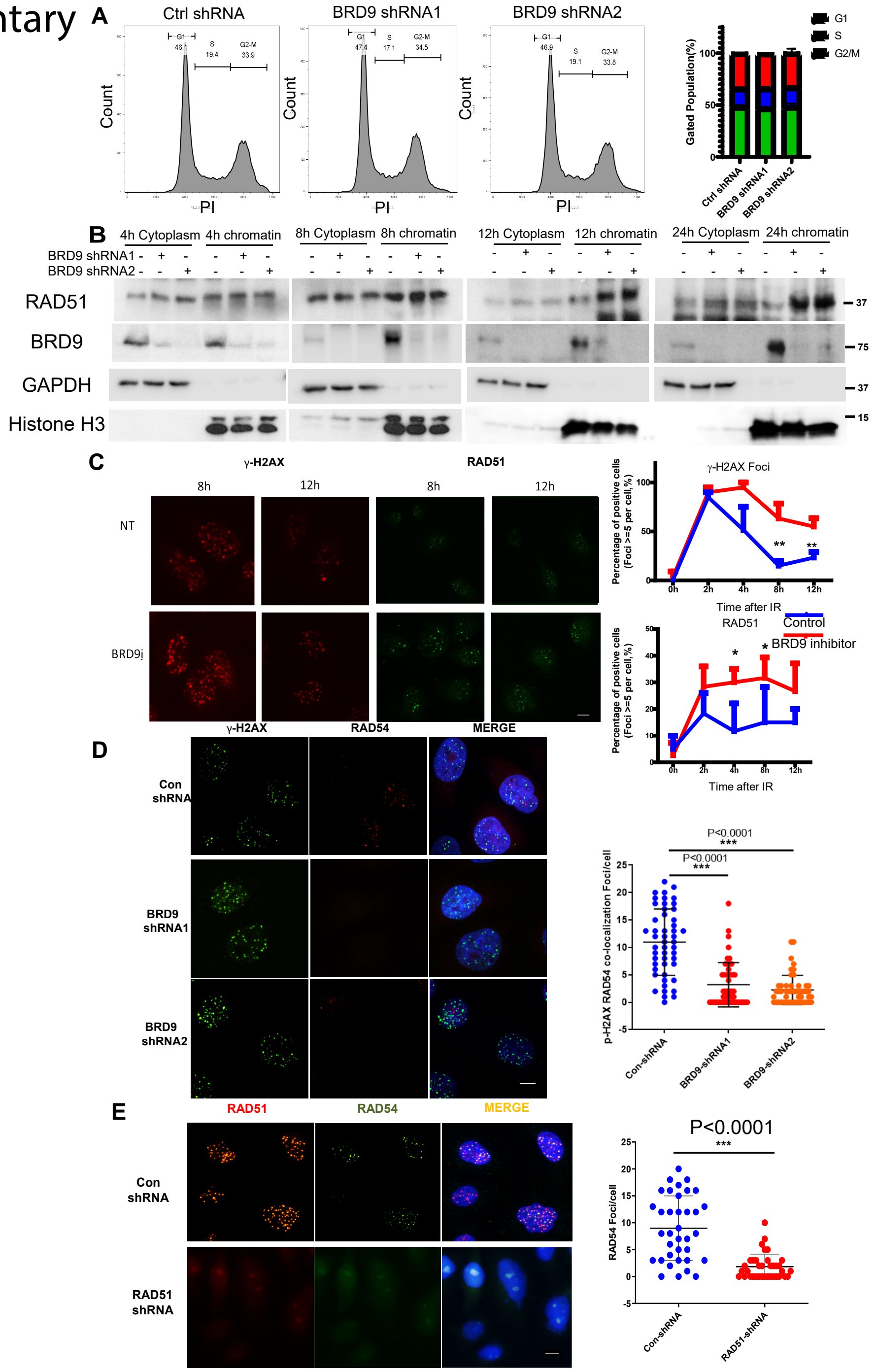

Supplementary Figure2. BRD9 does not affect cell cycle and is Required for the Co-localization of RAD51 and RAD54.

(A) OVCAR8 cells were infected with lentivirus expressing the indicated shRNA. Cells were harvested at the indicates time points, fixed and stained with PI. FACS analysis was performed. The fraction of cells in each phase of the cell cycle was quantified. Shown are the representative data (mean ± SEM) from three independent experiments.

(B) OVCAR8 cells were infected with lentivirus expressing the indicated shRNA. Cells were exposed to 10Gy IR and harvested at the indicated time points. Chromatin fraction extraction was performed. Blots were probed with the indicated antibodies.

(C) OVCAR8 cells were exposed to 10μM BRD9 inhibitor (BRD9i) for 24h., Cells were exposed to 2Gy IR and fixed at indicated time points. Cells were then stained with γ-H2AX (Red) and RAD51 (Green) antibodies. Shown are the representative images of γ-H2AX and RAD51 foci after the indicated treatments and indicated time following 2Gy irradiation (left). These foci were quantified (right). Shown are the representative data (mean ± SEM) from three independent experiments. *p<0.05,**p<0.01 by two-sided unpaired t-test Scale bar, 10 μm..

(D) OVCAR8 cells were infected with lentivirus expressing the indicated shRNA. OVCAR8 cells were exposed to 2Gy irradiation, 8 hours later, cells were stained with γ-H2AX and RAD54 antibodies . Shown are the representative images of γ-H2AX (green) and RAD54 (red) foci after the indicated treatments and 8h following 2Gy irradiation (left). This foci were quantified (right). Shown are the representative data (mean ± SEM) from three independent experiments. n=50 cells examined over 3 independent experiments. ***p<0.001 by two-sided unpaired t-test. Scale bar, 10 μm.

(E) OVCAR8 cells were infected with lentivirus expressing indicated shRNA. Cells were exposed to 2Gy IR. . 8 hours later, cells were stained with RAD51 and RAD54 antibodies. Shown are the representative images of γ-H2AX and RAD54 foci after the indicated treatments and 8h following 2Gy irradiation. This foci were quantified. Shown are the representative data (mean ± SEM) from three independent experiments. n=50 cells examined over 3 independent experiments. ***p<0.001 by two-sided unpaired t-test. Scale bar, 10 μm.

Supplementary Figure 3

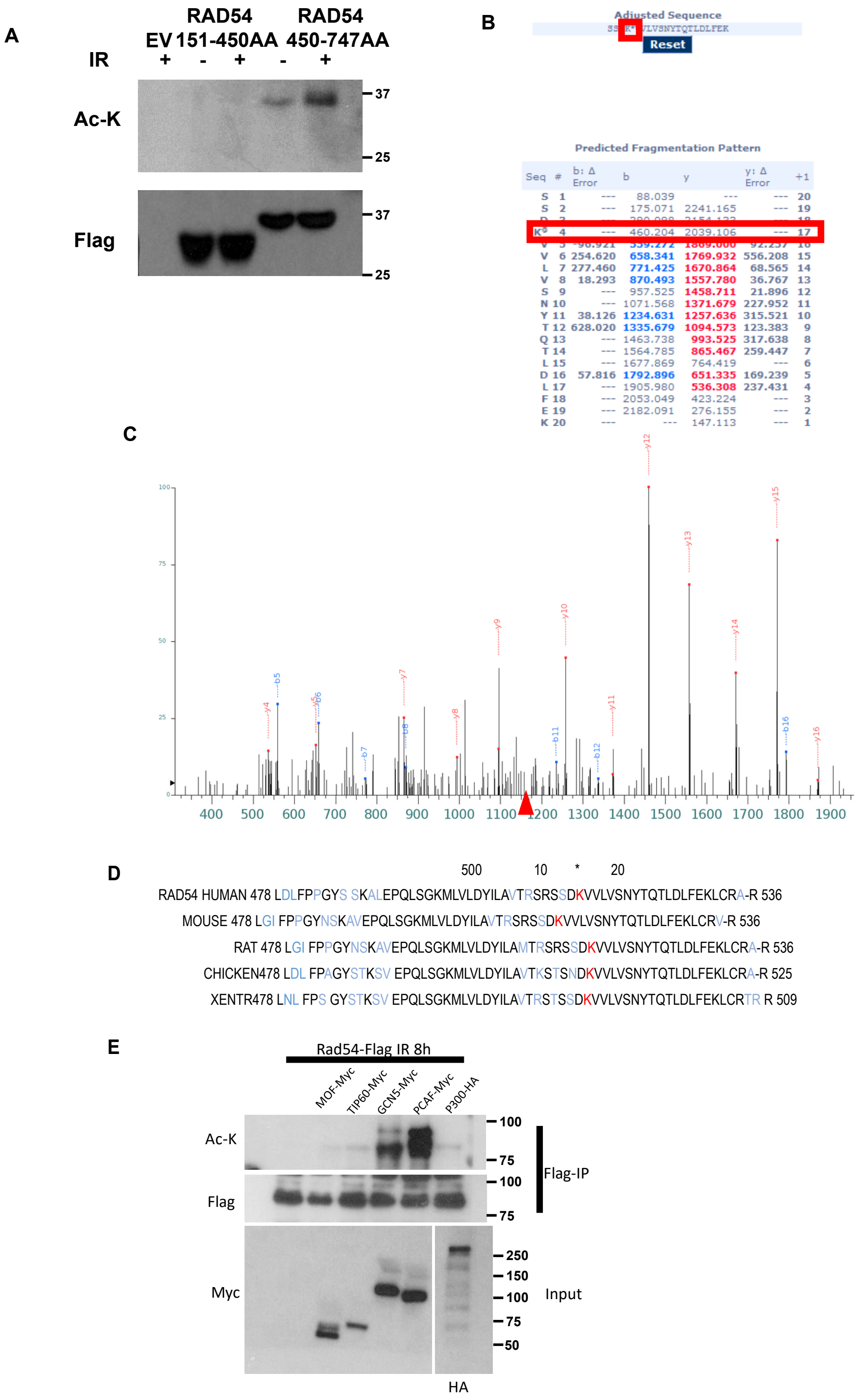

Supplementary Figure3. RAD54 is acetylated at K515 upon DNA Damage.

(A) 293T cells were transfected with indicated plasmids. 48h after transfection, cells were exposed to 10Gy IR. 8 hours later, cells were harvested. Immunoprecipitation with anti-Flag beads was performed. Blots were probed with the indicated antibodies.

(B-C) RAD54 protein was expressed in 293T cells and purified. Mass spectrometry was performed to map potential acetylation sites on RAD54 . (Marked by red circle)

(D) Conservation of the specific amino acid residues in RAD54 protein in various species. Numbers correspond to the positions of amino acid residues from N-terminus to C-terminus. Red color indicates the K515 site.

(E) 293T cells were transfected with indicated plasmids. 48h after transfection, cells were exposed to 10Gy IR. 8 hours later, cells were harvested. Immunoprecipitation with anti-Flag beads was performed. Blots were probed with the indicated antibodies.

Supplementary Figure 4

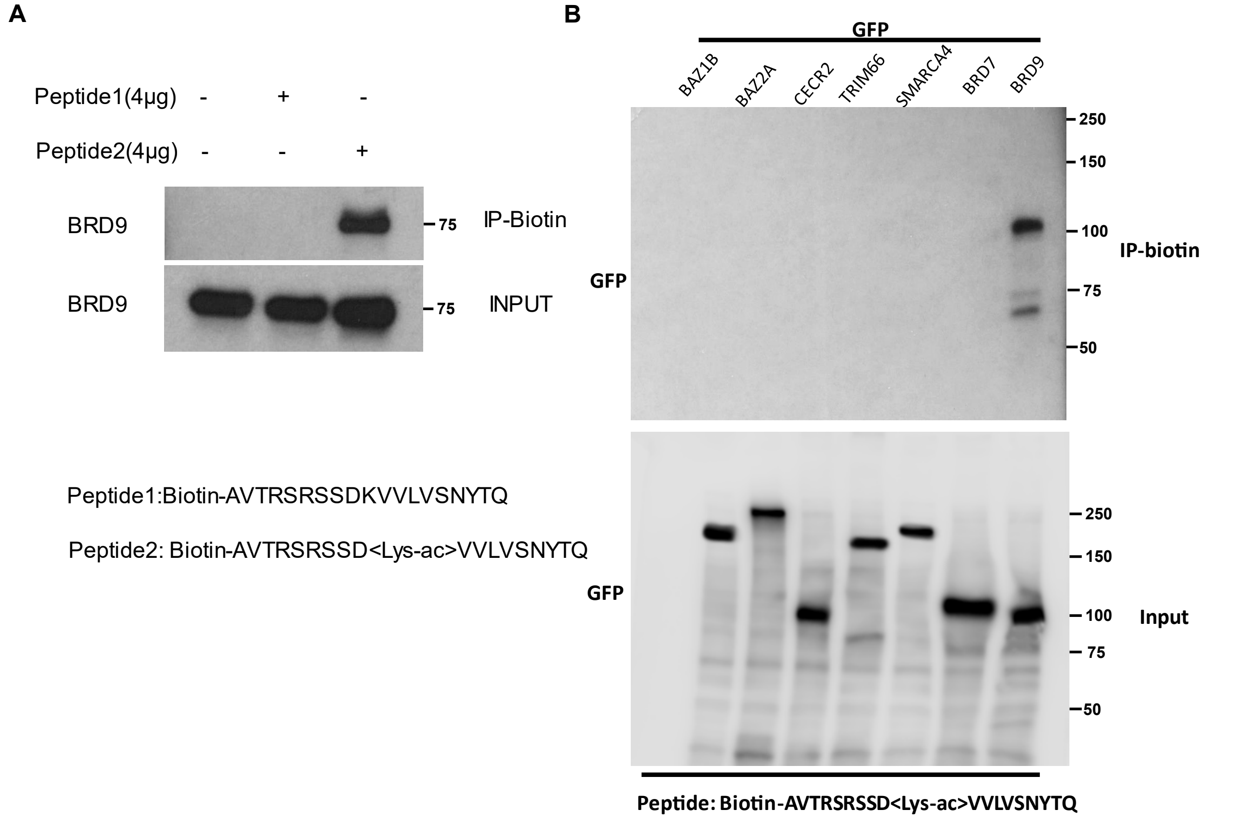

Supplementary Figure4. BRD9 can recognize RAD54 K515 peptide

(A) Lysates from 293T cells were incubated with RAD54 506-525AA peptide with/without acetylation on 515AA site. Immunoprecipitation with anti-Biotin beads was performed. Blots were probed with the indicated antibodies.

(B) 293T cells were transfected with indicated GFP-tagged plasmids. 48h after transfection, cells were harvested. Lysates were incubated with RAD54 506-525AA peptide with/without acetylation on 515AA site. Immunoprecipitation with anti-Flag beads was performed. Blots were probed with the indicated antibodies. .

Supplementary Figure 5

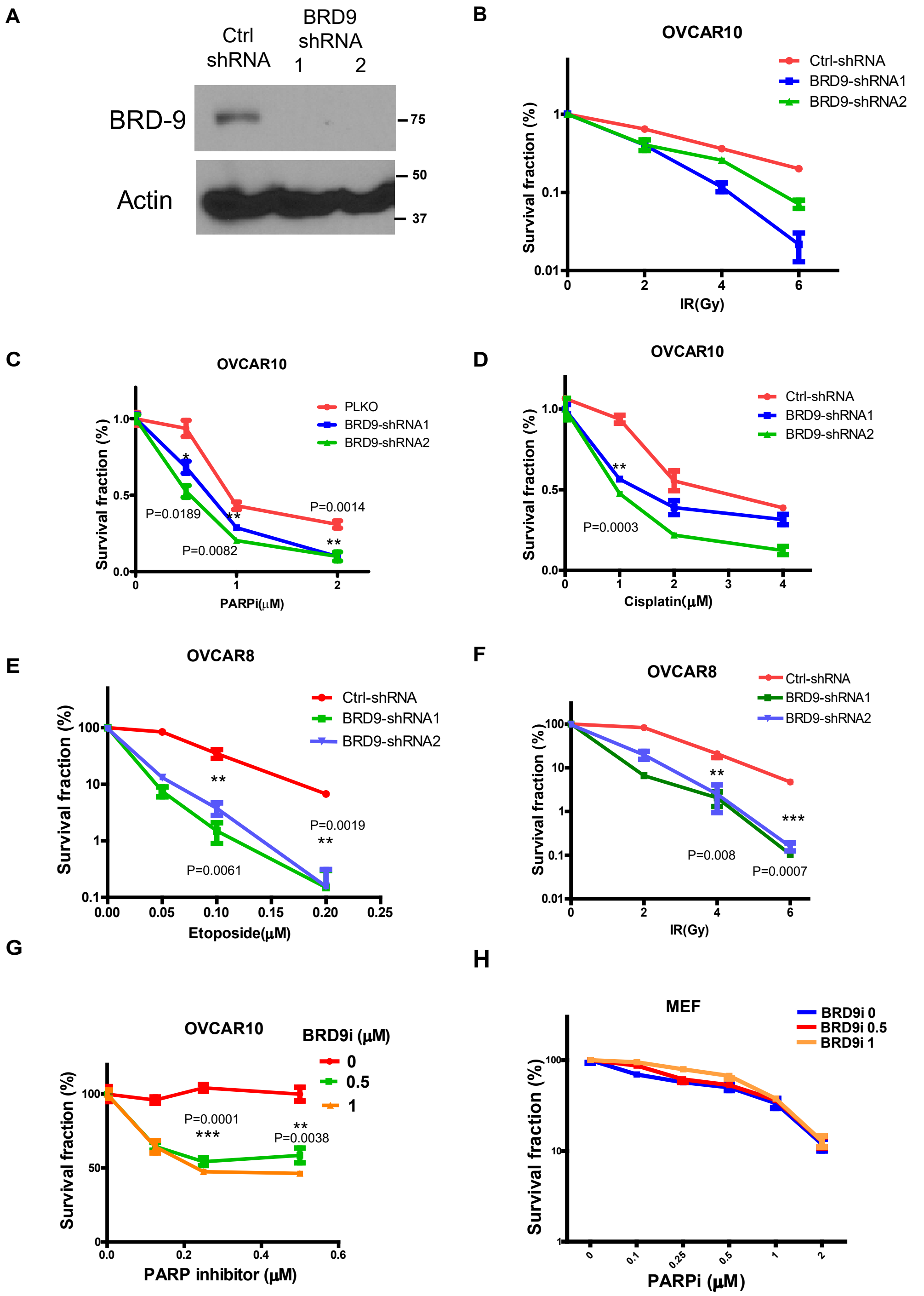

Supplementary Figure5. BRD9 is overexpressed in ovarian cancer and regulates response to DNA damaging agents.

(A-D) OVCAR10 cells were infected with lentivirus expressing Ctrl or BRD9 shRNA. Then cells were subjected to WB assay (A) to assess knockdown efficiency and colony formation assay to evaluate sensitivity to IR (B), Olaparib (C) and Cisplatin (D). Shown are the representative data (mean ± SEM) from three independent experiments. **p<0.01 by two-sided unpaired t-test.

(E) OVCAR8 cells were infected with lentivirus expressing Ctrl or BRD9 shRNA and subjected to colony formation assay to assess sensitivity to Etopside (E), and IR (F). Shown are the representative data (mean ± SEM) from three independent experiments. **p<0.01, ***p<0.001 by two-sided unpaired t-test.

(G) OVCAR10 cells were exposed to Olaparib (PARPi) and/or BRD9i and subjected to colony formation assay. Shown are the representative data (mean ± SEM) from three independent experiments.***p<0.001**p<0.01 by two-sided unpaired t-test.

(H) MEF cells were exposed to Olaparib (PARPi) and/or BRD9i and subjected to colony formation assay. Shown are the representative data (mean ± SEM) from three independent experiments by two-sided unpaired t-test.

Supplementary Figure 6

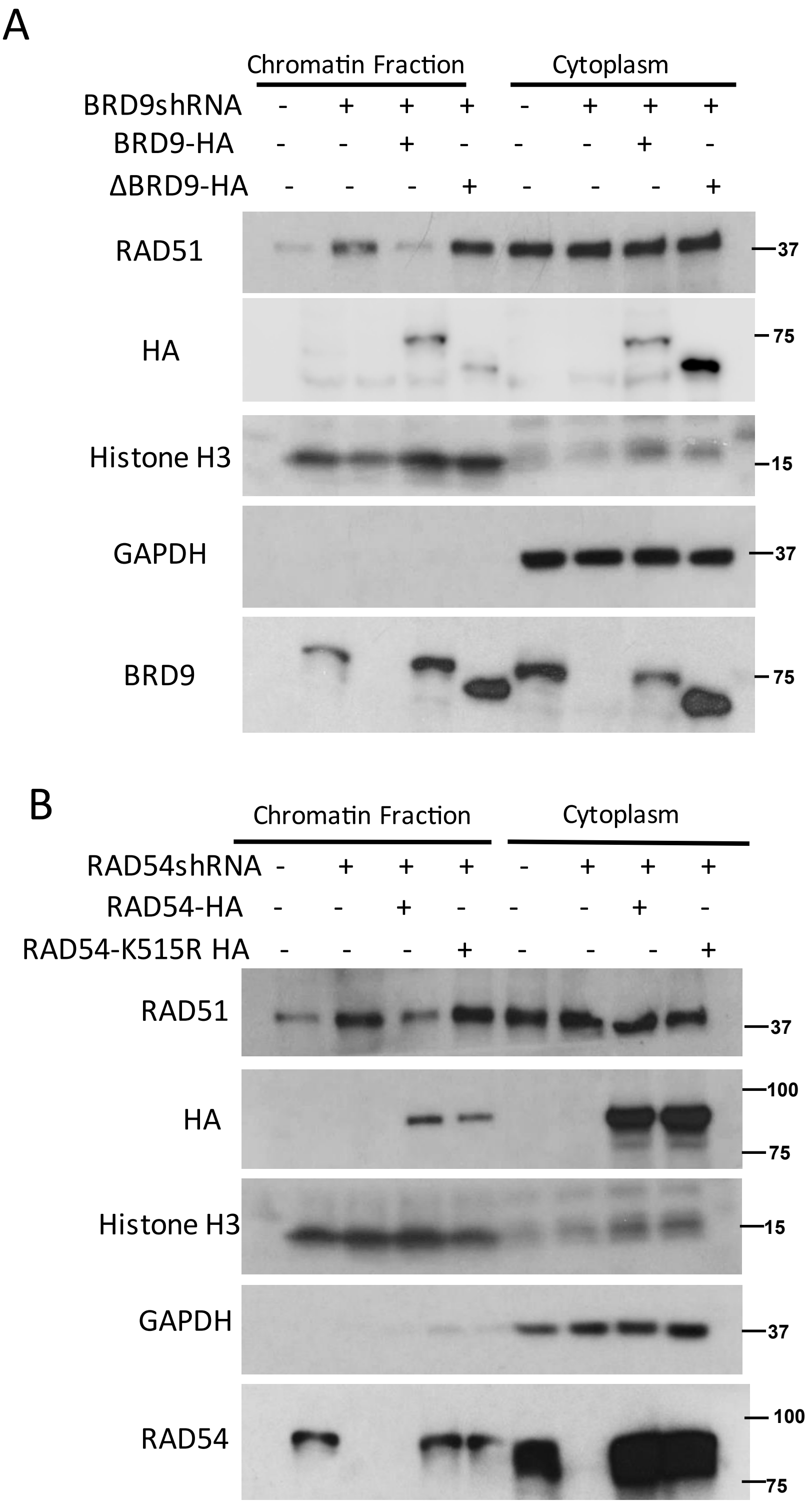

Supplementary Figure6. RAD51 retention on chromatin is induced by DNA damage in BRD9 bromodomain deletion mutant and RAD54 K515R mutant cells.

(A-B) 293T cells were infected with lentivirus expressing the indicated shRNA and the indicated constructs were expressed. Cells were exposed to IR (10Gy).Lysates were collected after 8 hours. Chromatin fraction was extracted and analyzed by Western blot. Blots were probed with the indicated antibodies.

Supplementary Figure 7

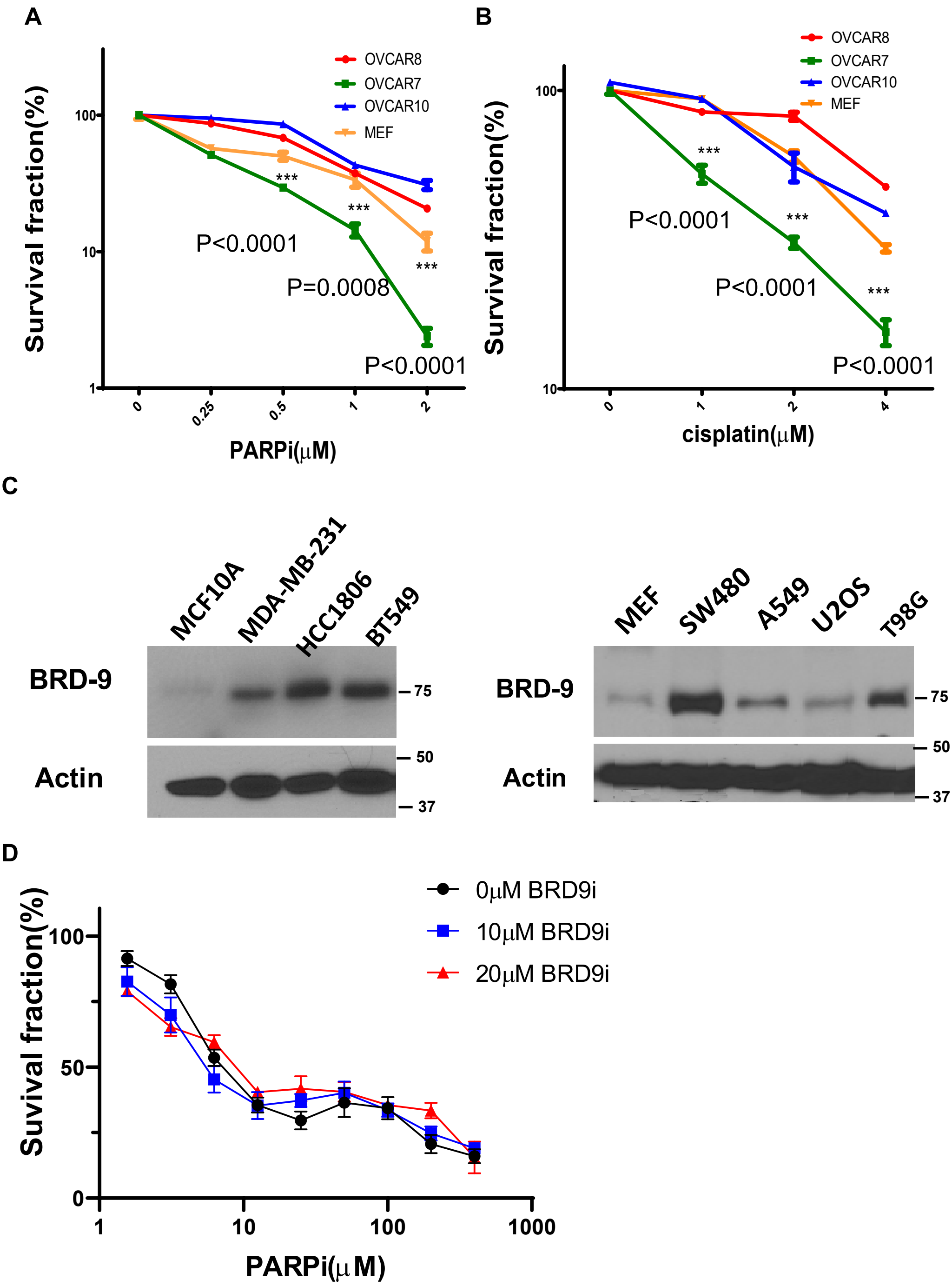

Supplementary Figure7. BRD9 expression of cancer cells is related to their sensitivity to DNA damaging therapy.

(A-B) OVCAR8, OVCAR7, OVCAR10 or MEF cells were subjected to colony formation assay to assess sensitivity to Olaparib (A), and Cisplatin (B). Shown are the representative data (mean ± SEM) from three independent experiments. ***p<0.001 by two-sided unpaired t-test ANOVA.

(C) Immunoblot of indicated proteins in indicated cancer cell lines.

(D). HOSE cells were treated with the indicated dose of PARPi and BRD9i.24h later, cells were subjected to MTS assay.

Supplementary Figure 8

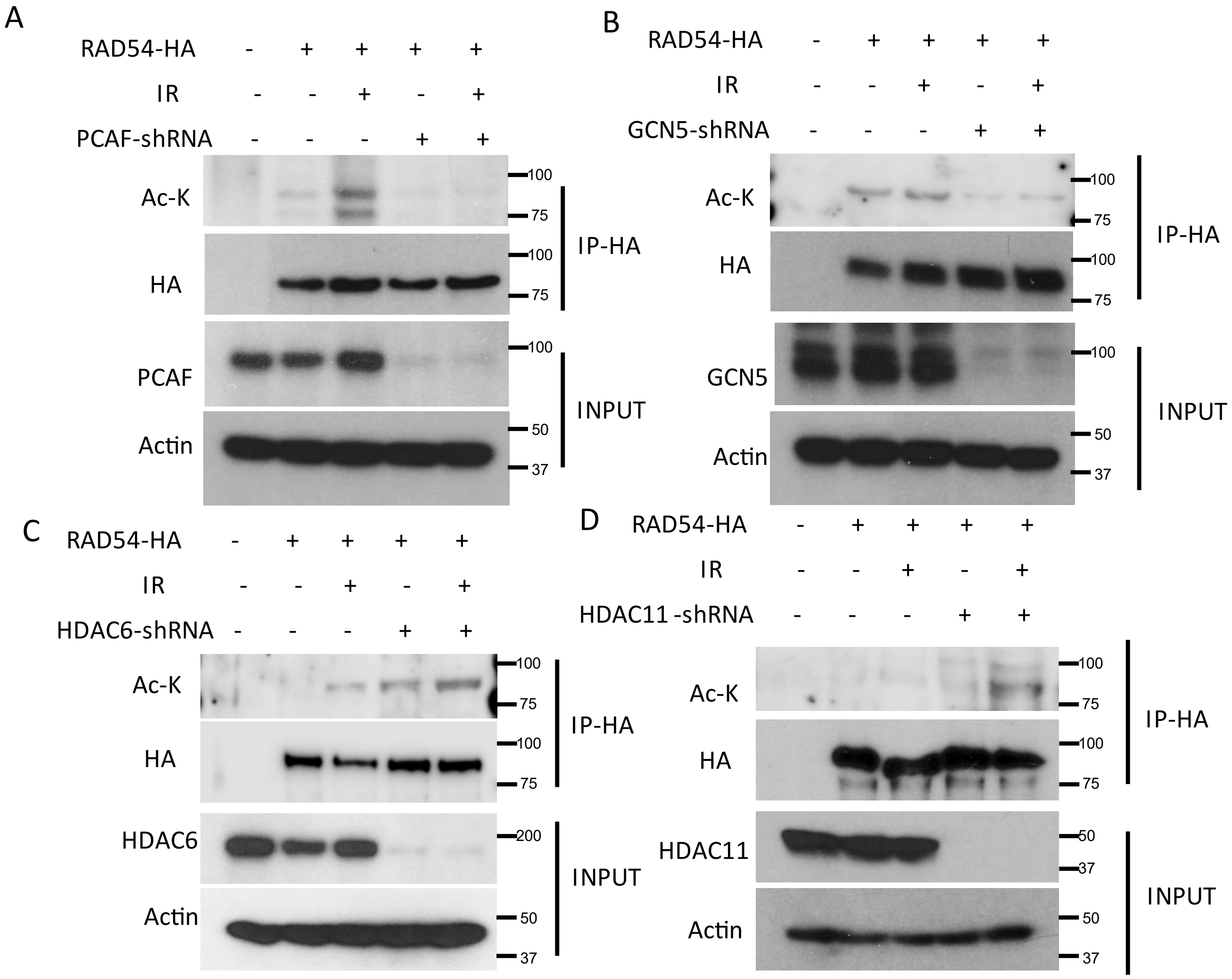

Supplementary Figure8. Knockdown of GCN5, PCAF and HDAC6 affects RAD54 acetylation after DNA damage.

(A-D) 293T cells were infected with lentivirus expressing PCAF (A), GCN5 (B), HDAC6 (C), or HDAC11(D) shRNA. Cells were transfected with the HA tagged RAD54 plasmid. 48h after transfection, cells were exposed to 10Gy IR and collected after 8h. Immunoprecipitation with anti-HA beads was performed. Blots were probed with the indicated antibodies.

Supplementary Figure 9

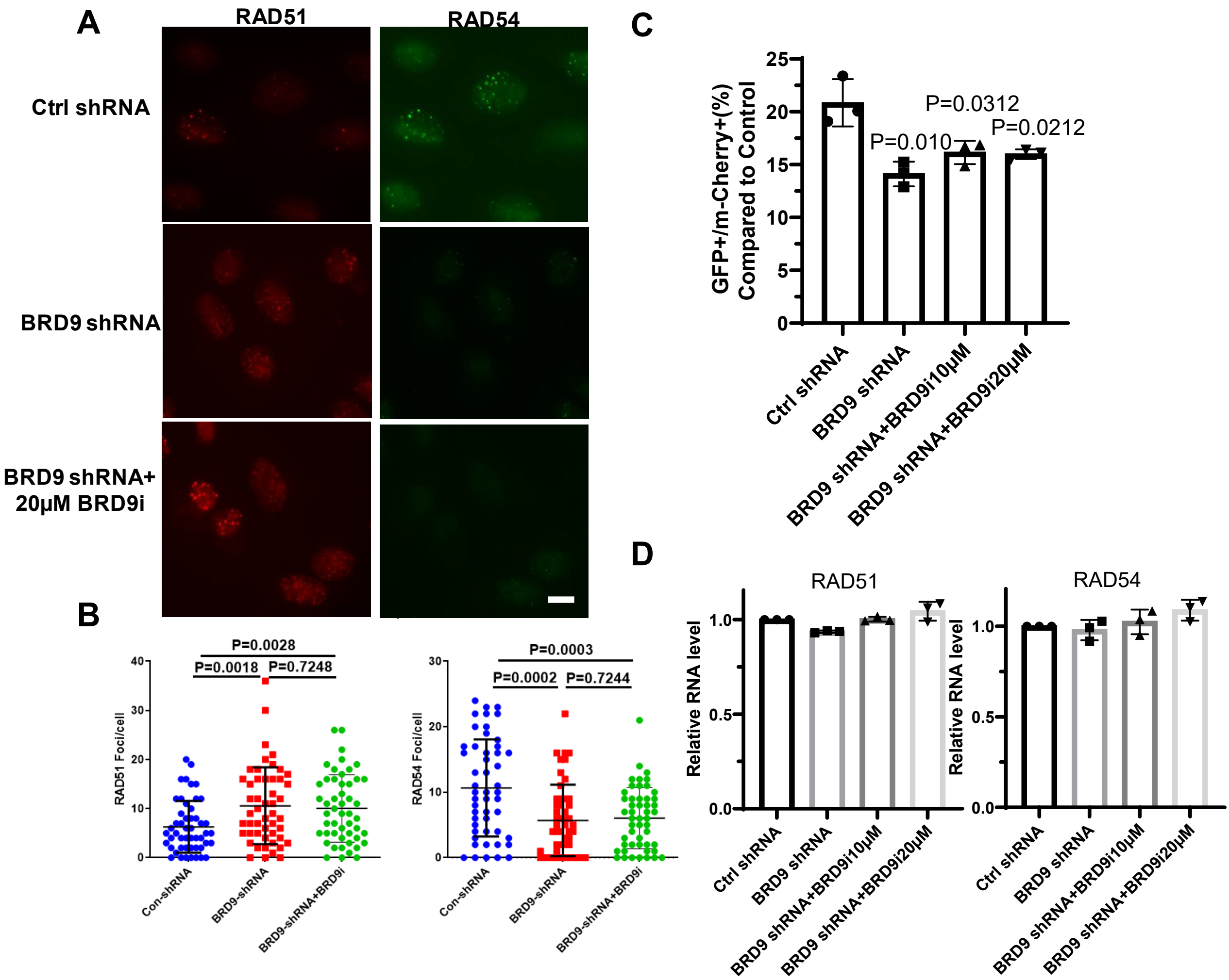

Supplementary Figure9. BRD9 inhibitor possess the anticipated on target effects.

(A-B) BRD9 inhibitor does not induce further reduction in RAD54 foci or greater retention of RAD51 foci on BRD9 knockdown OVCAR8 cells. Representative immunofluorescence images (A) and the quantification of foci (B) in cells infected with lentivirus expressing control (Ctrl) or BRD9 shRNA are shown. Cells were treated with BRD9 inhibitor 12h before 2Gy IR exposure. 8 hrs later, cells were stained with γ-RAD51 AND RAD54 antibodies. Shown are the representative data (mean ± SEM) from three independent experiments. n=50 cells examined over 3 independent experiments. P value were calculated byby two-sided unpaired t-test. NS, not significant. Scale bar, 10 μm.

(C). HR activity is not further inhibited by BRD9i in BRD9 knockdown 293T cells. Control (Ctrl) or BRD9 shRNA transfected 293T cells were treated with BRD9i. 12h later, repair capacity was assessed using reporter plasmids as outlined in the methods. Shown are the representative data (n=3, mean ± SEM) from three independent experiments. P value were calculated by by two-sided unpaired t-test.

(D) q-PCR assay demonstrating that RAD51 RAD54 expressions were not affected by either BRD9 knockdown using shRNA or BRD9 inhibitor treatment. OVCAR8 cells were transfected with BRD9 shRNA and treated with BRD9i as indicated. 12h later, the cells were subjected to q-PCR to assess RAD51 and RAD54 mRNA. Shown are the representative data (n=3, mean ± SEM) from three independent experiments.

Supplementary Figure 10

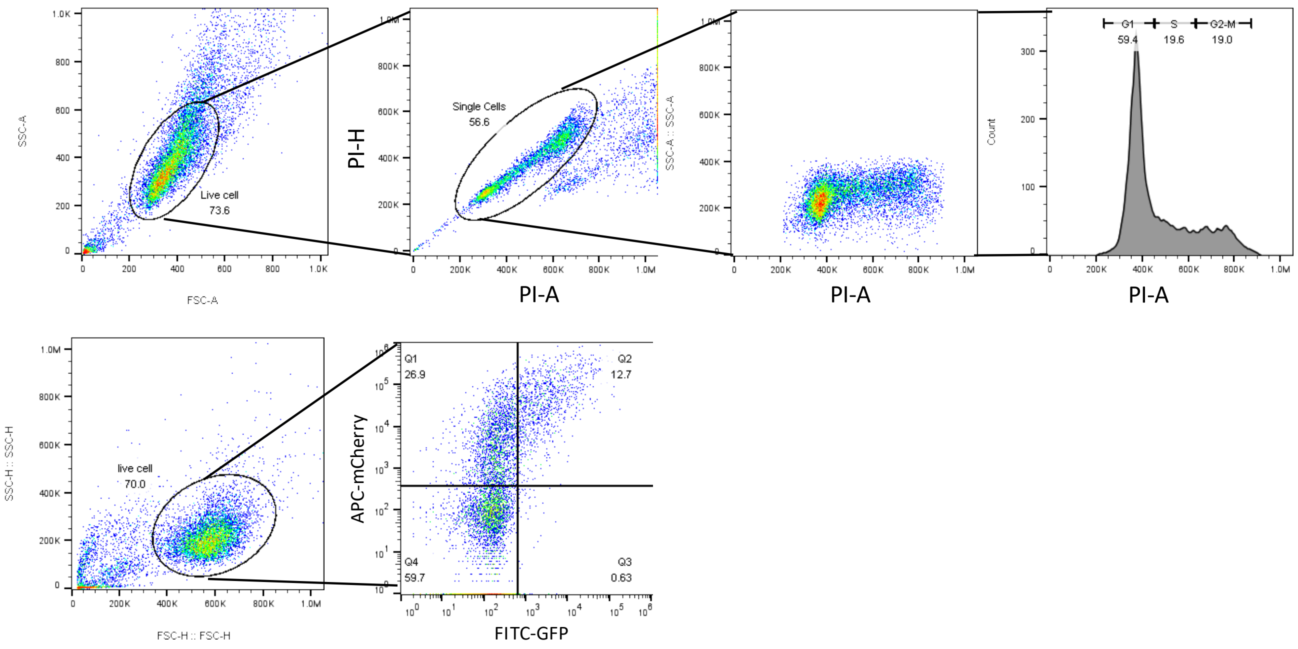

Supplementary Figure 10. (a) Gating strategy to determine the percentage of cells in each cell cycle (b) Gating strategy for HR/NHEJ assay, HR/NHEJ efficiency=Q2/(Q1+Q2)
