# Supplementary material for: The bromodomain containing protein BRD-9 orchestrates RAD51–RAD54 complex formation and regulates homologous recombination-mediated repair
Source: Nat Commun. 2020 May 26;11:2639. doi: 10.1038/s41467-020-16443-x (PMC7251110; doi:10.1038/s41467-020-16443-x)
Supplement: Supplementary file 3 — Reporting Summary [file 41467_2020_16443_MOESM3_ESM.pdf]

nature research

Corresponding author(s): Jian Yuan, Robert Mutter

Last updated by author(s): Apr 5, 2020

Reporting Summary

Nature Research wishes to improve the reproducibility of the work that we publish. This form provides structure for consistency and transparency in reporting. For further information on Nature Research policies, see Authors & Referees and the Editorial Policy Checklist.

Statistics

For all statistical analyses, confirm that the following items are present in the figure legend, table legend, main text, or Methods section.

n/a	Confirmed	
☐	☒	The exact sample size (*n*) for each experimental group/condition, given as a discrete number and unit of measurement
☐	☒	A statement on whether measurements were taken from distinct samples or whether the same sample was measured repeatedly
☐	☒	The statistical test(s) used AND whether they are one- or two-sided<br>*Only common tests should be described solely by name; describe more complex techniques in the Methods section.*
☐	☒	A description of all covariates tested
☐	☒	A description of any assumptions or corrections, such as tests of normality and adjustment for multiple comparisons
☐	☒	A full description of the statistical parameters including central tendency (e.g. means) or other basic estimates (e.g. regression coefficient) AND variation (e.g. standard deviation) or associated estimates of uncertainty (e.g. confidence intervals)
☒	☐	For null hypothesis testing, the test statistic (e.g. *F*, *t*, *r*) with confidence intervals, effect sizes, degrees of freedom and *P* value noted<br>*Give P values as exact values whenever suitable.*
☒	☐	For Bayesian analysis, information on the choice of priors and Markov chain Monte Carlo settings
☒	☐	For hierarchical and complex designs, identification of the appropriate level for tests and full reporting of outcomes
☒	☐	Estimates of effect sizes (e.g. Cohen's *d*, Pearson's *r*), indicating how they were calculated

Our web collection on statistics for biologists contains articles on many of the points above.

Software and code

Policy information about availability of computer code

Data collection	Attune NxT v2.6
Data analysis	GraphPad Prism7, Microsoft Excel,flowjo

For manuscripts utilizing custom algorithms or software that are central to the research but not yet described in published literature, software must be made available to editors/reviewers. We strongly encourage code deposition in a community repository (e.g. GitHub). See the Nature Research guidelines for submitting code & software for further information.

Data

Policy information about availability of data

All manuscripts must include a data availability statement. This statement should provide the following information, where applicable:

- Accession codes, unique identifiers, or web links for publicly available datasets
- A list of figures that have associated raw data
- A description of any restrictions on data availability

All the data are available from the corresponding authors upon reasonable request. Raw data for figures has been listed in Supplemental figures and Supplemental informations.

Field-specific reporting

Please select the one below that is the best fit for your research. If you are not sure, read the appropriate sections before making your selection.

☒ Life sciences ☐ Behavioural & social sciences ☐ Ecological, evolutionary & environmental sciences

Life sciences study design

All studies must disclose on these points even when the disclosure is negative.

Sample size	No statistical method was used to predetermine sample size. All experiments were included with multiple biological replicates based on previous experiences.
Data exclusions	No samples were excluded from analysis.
Replication	All results were tested and confirmed with at least three independent experiments.
Randomization	Samples were randomly allocated into experimental groups prior to treatment. For example, since most of our experiments involved cells, plates were randomly allocated to groups.
Blinding	No blinding assessment was performed as microscopy and FACS data. For manual microscopy, Data were collected randomly choose at least 5 region. For FACs and high-content microscopy data were anlayzed automatically. One individual performed the experiment while another individual (blinded to the group allocation) performed the analysis.

Reporting for specific materials, systems and methods

We require information from authors about some types of materials, experimental systems and methods used in many studies. Here, indicate whether each material, system or method listed is relevant to your study. If you are not sure if a list item applies to your research, read the appropriate section before selecting a response.

Materials & experimental systems

n/a	Involved in the study	
☐	☒	Antibodies
☐	☒	Eukaryotic cell lines
☒	☐	Palaeontology
☐	☒	Animals and other organisms
☒	☐	Human research participants
☒	☐	Clinical data

Methods

n/a	Involved in the study	
☒	☐	ChIP-seq
☐	☒	Flow cytometry
☒	☐	MRI-based neuroimaging

Antibodies

Antibodies used

Anti-BRD9 (rabbit)for IF (1:500) were homemade.
Anti-53BP1(rabbit)for IF (1:500) were homemade.
Anti-BRD9(A303-781A rabbit) for WB(1:1000) was purchased from Bethyl Laboratories.
Anti-RAD54(sc-166370 mouse) for WB(1:1000) were purchased from Santa Cruz Biotechnology.
Anti-BRCA1(sc-6954 mouse) for IF(1:1000) were purchased from Santa Cruz Biotechnology.
Anti-RAD51antibody(sc-377467 mouse) for WB(1:1000) were purchased from Santa Cruz Biotechnology.
Anti-Ack antibody (600-401-939 rabbit) for WB (1:500)was purchased from Rockland.
Anti-γ H2AX (05-636 mouse) for IF (1:1000) was purchased from Millipore.
Anti-RAD51(ab133534 rabbit) for IF (1:500) were purchased from Abcam
Anti-RAD54(ab11055 mouse) for IF (1:500) were purchased from Abcam.
anti-Myc 1:2000 (sc-40, mouse) for WB(1:1000) were purchased from Santa cruz.
Anti-Flag(F1804 mouse) for WB(1:1000) were purchased from Sigma.
anti-HA(H9658 mouse) for WB(1:1000) were purchased from Sigma.
Anti-β -Actin(A1978 mouse) for WB(1:1000) were purchased from Sigma.
Anti SMARCC1(11956 rabbit) for WB(1:1000) were purchased from Cell Signaling Technology.
Anti BRG1(3508 rabbit) for WB(1:1000) were purchased from Cell Signaling Technology.
Anti BAF250(12354 rabbit) for WB(1:1000) were purchased from Cell Signaling Technology.
Anti BRM(11966 rabbit) for WB(1:1000) were purchased from Cell Signaling Technology.
Anti-H3 (17168-1-AP, rabbit) for WB(1:1000) were purchased from Proteintech.
Alexa Fluor® 488 AffiniPure Donkey Anti-Rabbit IgG (H+L 715-585-150) Alexa Fluor® 594 AffiniPure Donkey Anti-Rabbit IgG (H+L 711-585-152) Alexa Fluor® 488 AffiniPure Donkey Anti-Mouse IgG (H+L 715-545-150) and Alexa Fluor® 594 AffiniPure Donkey Anti-Mouse IgG (H+L 715-585-150) were purchased from Jackson Lab.
Donkey Anti-Mouse IgG (H+L) ML*(715-675-151) and Donkey Anti-Rabbit IgG (H+L) ML*(711-675-152) were purchased from Jackson ImmunoResearch.

Validation

Homemade antibodies were validated by knockdown cells or comparing with other manufactures antibodies. other antibodies were validated by the manufactures.

Eukaryotic cell lines

Policy information about cell lines

Cell line source(s)	HTC116(ATCC)<br>HEK293T (ATCC)<br>OVCAR8(ATCC)<br>MEF(ATCC)<br>U2OS (ATCC)<br>OVCAR7(ATCC)<br>OVCAR10(ATCC)<br>A2780(ATCC)<br>MCF10A(ATCC)<br>MDA-MB-231(ATCC)<br>HCC1806(ATCC)<br>BT549(ATCC)<br>T98G(ATCC)<br>SW480(ATCC)<br>SKOV3(Thermo Fisher Scientific)<br>EFO-27(DSMZ company)<br>Human Ovarian epithelial cells(ScienCell)
Authentication	The Cell lines have been authenticated based on morphological criteria.
Mycoplasma contamination	All cell lines were tested negative for mycoplasma.
Commonly misidentified lines<br>(See ICLAC register)	No commonly misidentified cell lines were used.

Animals and other organisms

Policy information about studies involving animals; ARRIVE guidelines recommended for reporting animal research

Laboratory animals	Outbred athymic nude mice. 6-18 week old and female mice are used in experiments.
Wild animals	No wild animals were used in this study.
Field-collected samples	No filed-collected samples were used in this study.
Ethics oversight	All animal work was approved by the Institutional Animal Care and Use Committee (IACUC) under protocol A00002864-17

Note that full information on the approval of the study protocol must also be provided in the manuscript.

Flow Cytometry

Plots

Confirm that:

☒ The axis labels state the marker and fluorochrome used (e.g. CD4-FITC).

☒ The axis scales are clearly visible. Include numbers along axes only for bottom left plot of group (a 'group' is an analysis of identical markers).

☒ All plots are contour plots with outliers or pseudocolor plots.

☒ A numerical value for number of cells or percentage (with statistics) is provided.

Methodology

Sample preparation	We used FACS for cell cycle analysis (cell cycle and sub G1) or HR/NHEJ analysis. Cells were transfected with m-cherry/GFP expression plasmids or PI , trypsinized and suspended in PBS. Further details of the experimental procedures are provided in the materials and Methods.
Instrument	Attune NxT Flow Cytometer (Thermo fisher SCIENTIFIC)
Software	Attune NxT Flow Cytometer software v2.6
Cell population abundance	No flow based sorting was performed.

Gating strategy

The FSC/SSC gates defined the single cell population and gated indicated antibodies positive population. For cell cycle, after FSC/SSC gating, PI-A and PI-H were gated for single cell population and analyzed PI-A positive population as cell cycle distribution.

☒ Tick this box to confirm that a figure exemplifying the gating strategy is provided in the Supplementary Information.
